# Simulation of Long-Term Carbon and Nitrogen Dynamics in Grassland-Based Dairy Farming Systems to Evaluate Mitigation Strategies for Nutrient Losses

**DOI:** 10.1371/journal.pone.0067279

**Published:** 2013-06-27

**Authors:** Ghulam Abbas Shah, Jeroen C.J. Groot, Ghulam Mustafa Shah, Egbert A. Lantinga

**Affiliations:** Farming Systems Ecology Group, Wageningen University, Wageningen, The Netherlands; University of Florida, United States of America

## Abstract

Many measures have been proposed to mitigate gaseous emissions and other nutrient losses from agroecosystems, which can have large detrimental effects for the quality of soils, water and air, and contribute to eutrophication and global warming. Due to complexities in farm management, biological interactions and emission measurements, most experiments focus on analysis of short-term effects of isolated mitigation practices. Here we present a model that allows simulating long-term effects at the whole-farm level of combined measures related to grassland management, animal housing and manure handling after excretion, during storage and after field application. The model describes the dynamics of pools of organic carbon and nitrogen (N), and of inorganic N, as affected by farm management in grassland-based dairy systems. We assessed the long-term effects of delayed grass mowing, housing type (cubicle and sloping floor barns, resulting in production of slurry and solid cattle manure, respectively), manure additives, contrasting manure storage methods and irrigation after application of covered manure. Simulations demonstrated that individually applied practices often result in compensatory loss pathways. For instance, methods to reduce ammonia emissions during storage like roofing or covering of manure led to larger losses through ammonia volatilization, nitrate leaching or denitrification after application, unless extra measures like irrigation were used. A strategy of combined management practices of delayed mowing and fertilization with solid cattle manure that is treated with zeolite, stored under an impermeable sheet and irrigated after application was effective to increase soil carbon stocks, increase feed self-sufficiency and reduce losses by ammonia volatilization and soil N losses. Although long-term datasets (>25 years) of farm nutrient dynamics and loss flows are not available to validate the model, the model is firmly based on knowledge of processes and measured effects of individual practices, and allows the integrated exploration of effective emission mitigation strategies.

## Introduction

During the last century, in many agricultural systems the inputs of nitrogen (N) bound by the Haber-Bosch process have largely replaced N from sources like symbiotic fixation and mineralization from manures, crop residues and soil organic matter [1]. The large amount of artificial fertilizers used in agroecosystems has resulted in high concentrations of reactive N in the biosphere, which caused negative effects on soil, water and air quality with detrimental consequences for ecosystems, food supply chains and human health [2], [3]. Large improvements have been reached in the environmental performance of dairy farming systems through improved farming practices, underpinning research and supporting policies since the 1980s. Nevertheless, effective integrated approaches to reduce these negative effects of agriculture are still urgently needed.

Flows of N on grassland-based dairy and mixed crop-livestock farming systems can be conceptualized as a cycle from soil N uptake by grassland and crops, which are supplied to animals as feed, the ingested feed is partly incorporated into products but the largest proportion is excreted, and the excreta can be used to fertilize the soil [4]–[7]. Gaseous emissions and losses to soil and water can occur at various points in the N cycle, and increase when the total amount of N cycling in the system is enhanced by larger inputs [8]. Therefore, many environmental policies have focused on reduction of inputs to decrease the amount of N cycling in the farming system [9], [10]. When artificial N inputs are diminished, or even completely abandoned as in organic farming systems, the dependence on natural sources of N increases. Then management should focus more on incorporation of legumes like clovers to fix atmospheric N_2_, cropping and animal housing systems that optimize crop residue and manure utilization, and on slow processes of build-up of organic matter (OM) and N stocks in soils [11]. The interactions among these biological processes are complex and prone to environmental variability, and as a consequence farmers often struggle to develop a coherent new management strategy at lower input levels [10].

At a given level of N cycling, N use efficiency can be increased through mitigation practices. Many policies and practices have been developed to reduce losses from manure management chains for slurry and solid cattle manure (SCM). In the Netherlands, farms with cubicle housing of livestock that produce slurry are obliged to use covered slurry storage facilities and should apply the slurry into the soil to reduce exposure to air and concomitant losses [12]. In contrast, when animals are housed in deep litter or sloping floor barns, a mixture of faeces, urine and bedding material (mostly wheat straw) is produced. For such straw-based systems, Shah et al. [13], [14] showed that application of bedding additives like zeolite, farm topsoil and lava meal inside the barn have potential to reduce N losses and enhance N utilization by crops. In another study, Shah et al. [15] reported that anaerobic storage of solid manure followed by 10 mm of irrigation immediately after its application diminished ammonia (NH_3_) emission rate by 92% while herbage apparent N recovery increased by 33% as compared to non-irrigated manure.

However, identification of appropriate measures to apply on farms in order to reduce losses is complicated because reduction of losses at one point in the N cycle by a mitigating practice are often compensated by higher losses at other points in the cycle [16], [17]. Moreover, the consequences of adjustments to farming practices in the long term should be evaluated. To avoid compensatory loss pathways and negative impacts in the long run, a systems-oriented analysis of the whole farm and the N cycle is needed to construct a coherent long-term strategy of mitigation of losses [18], [19]. Simulation models can support the evaluation of measures and the development of effective strategies (see [20] for a review of models of livestock systems). Here, we employ an extended version of the Farm DANCES eco-mathematical model [9], [21] to evaluate and compare the long-term productive, environmental and economic performance of dairy systems utilizing different manure types and loss mitigation practices. The model simulates the dynamics of organic carbon (C) and N and of inorganic N available for plant uptake on grassland-based dairy farms. It quantifies mineralization and immobilization, NH_3_ volatilization and combined soil N losses (through runoff, leaching and denitrification). Farm management decisions on grassland management, the type of manures produced, adjustment of storage methods, and use of low-emission techniques influence these processes at various points of N cycle.

The objectives of the current study were (i) to explore long-term effects of adapting NH_3_ mitigation practices such as use of manure bedding additives, contrasting manure storage methods and irrigation after application of covered manure, on the time course of soil organic C and N contents, soil N mineralization, farm productivity and economics, (ii) to compare effects of strategic adjustments of solid cattle manure and slurry-based systems on farm performance, and (iii) to define a coherent strategy combining effective practices, to mitigate losses and to improve farm productive, environmental and economic performance.

### Model Description

**Figure 1:**
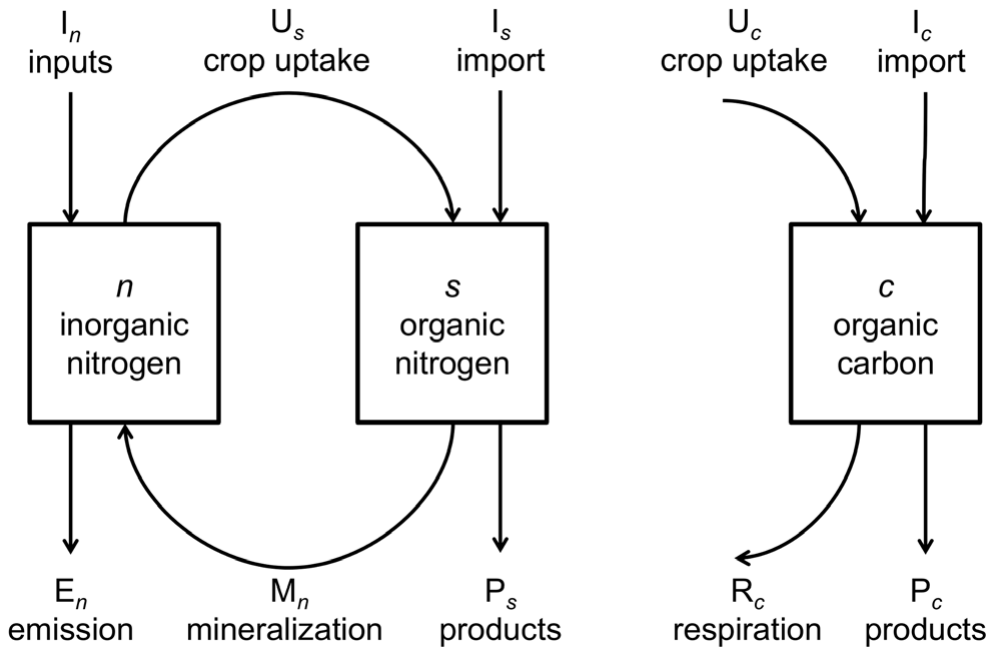
State variables of the model: organic carbon and nitrogen (*c* and *s*), and available inorganic nitrogen (*n*). **The arrows indicate flows between the pools.** ****

The model simulates the dynamics of three state variables that quantify the amount of organic carbon (*c*) and nitrogen (*s*), and the integrated amount of inorganic nitrogen that is available for plant uptake throughout the year (*n*). The state variables and the aggregate flows of N and C on the farm are presented in Fig. 1. Hence, the differential equations of the model are:
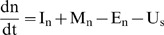
(1)

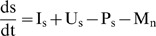
(2)

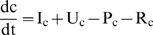
(3)Where

*I_n_* = inputs of inorganic nitrogen from fertilizers, deposition and fixation (kg ha^–1^ year^–1^).

*M_n_* = mineralization of organic nitrogen (kg ha^–1^ year^–1^).

*E_n_* = losses of inorganic nitrogen through NH_3_ volatilization and soil N losses by leaching, runoff and denitrification (kg ha^–1^ year^–1^).

*U_s_* = net uptake of inorganic nitrogen into organic material by plants, corrected for mineralization from decay of plant biomass, manure and animal digestion (kg ha^–1^ year^–1^).

*I_s_* = inputs of organic nitrogen in feeds (kg ha^–1^ year^–1^).

*P_s_* = export of organic nitrogen in crops, manure and animal products (kg ha^–1^ year^–1^).

*I_c_* = inputs of organic carbon in feeds (kg ha^–1^ year^–1^).

*U_c_* = net uptake of organic carbon into organic material by plants, corrected for respiration from decay of manure and animal digestion (kg ha^–1^ year^–1^).

*P_c_* = export of organic carbon in crops, manure and animal products (kg ha^–1^ year^–1^).

*R_c_* = respiration of organic carbon through decay by soil biota (kg ha^–1^ year^–1^).

The model is target-oriented, based on a production level of milk and meat that is defined by the size and productivity of the herd (see Table S1 for parameter values). Energy and protein requirements were calculated on the basis of the Dutch feed evaluation systems [22], [23]. The animals are fed with on-farm produced grass and feed crop products (in this case silage maize), and supplementary feed is imported when the amount of feeds produced on the farm is insufficient to cover the energy and protein requirements of the herd. If there is a surplus of on-farm produced feed crop export occurs.

**Figure 2:**
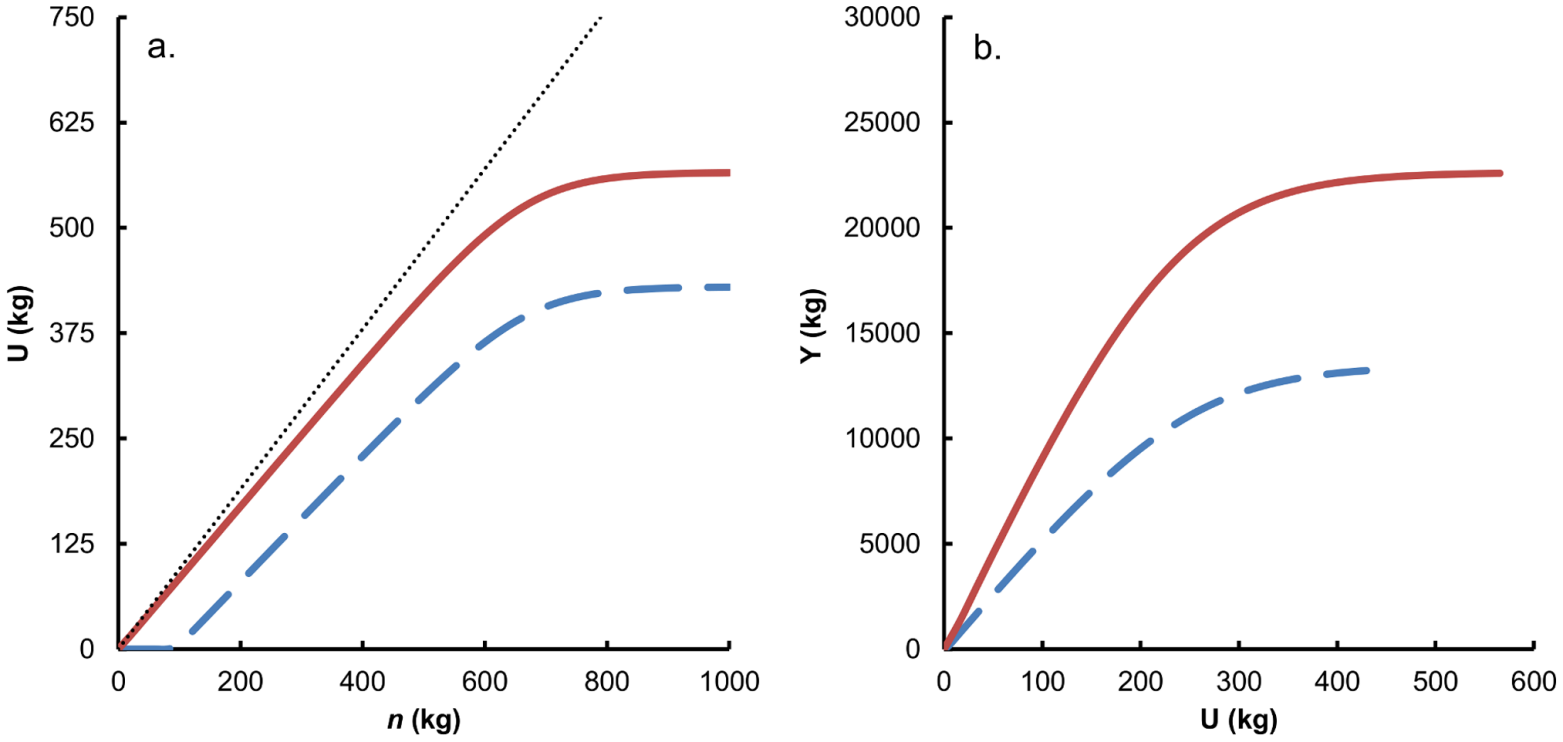
Relation between available inorganic N (*n*), uptake by the grassland (U) and biomass production (Y) for total (solid line) and harvested (dashed line) biomass. The dotted line indicates the annual withdrawal of inorganic N, N soil losses are calculated as the difference between this line and N uptake in total biomass in a.

Grassland production is described by the response of N uptake (U) to available inorganic nitrogen (*n*), and the relation between U and biomass yield (Y) (Fig. 2). These relations are defined by adjusted expo-linear equations [9], [24]. N is taken up in harvestable and unharvested biomass, because farm animals can only harvest part of the total amount of plant biomass produced, the remainder staying behind in the field as organic material. Therefore, we distinguished total and harvested amounts of N uptake (U_T_ and U_H_) and total and harvested biomass (Y_T_ and Y_H_), which resulted in four equations. Equation (4) shows the general form of the expo-linear equation.
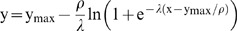
(4)Where

*y* = the dependent variable, representing U_T_, Y_T_, U_H_ or Y_H_ (kg ha^–1^ year^–1^).

*x* = the independent variable, representing *n*, U_T_, or U_H_ (kg ha^–1^ year^–1^).

*y_MAX_* = maximum value of y, representing U_MAX,T_, Y_MAX,T_, U_MAX,H_ or Y_MAX,H_ (kg ha^–1^ year^–1^).

*ρ* = initial response of y to x, representing ρ_U,T_, ρ_Y,T_, ρ_U,H_ and ρ_Y,H_ (kg kg^–1^ ha^–1^ year^–1^).

*λ* = the decline of the response of y to x (kg^–1^).

The initial response of N uptake to available inorganic N and of biomass production to N uptake is linear, with an initial slope ρ. This initial slope declines with a rate depending on λ until the maximum y_MAX_ is reached. The ratio between U_MAX,H_ and U_MAX,T_ is denoted h_N_: the fraction of harvested N in biomass. Maximum dry matter yield is calculated as Y_MAX_ = Y_MAX_/α_MAX_, where α_MAX_ is the maximum N content of grass. The initial response of both total and harvested plant biomass yield to N uptake is calculated from the minimum N content in herbage: ρ_Y_ = 1/α_MIN_, with α_MIN_ as the minimum N content of grass. The grassland production curves were calibrated for mixed use by mowing and grazing. For silage maize a yield level Y_MAIZE_ is defined. The parameters for maize production and the grassland production curves can be found in Table S2.

The harvested biomass is fed to animals and can be partly exported in case of a feed surplus. The feed is partly digested by the animals (k_D_, year^–1^) and the undigested fraction enters the manure, where it is subjected to further degradation (k_M_, year^–1^) during storage before application to the field. Manure degradability is dependent on the feed quality, therefore the k_M_ is proportional to k_D_ using constant g_M_, so that k_M_ = k_D_•g_M_. The parameters regarding feed quality are in Table S3.

Soil processes include degradation and additions of OM, and soil N losses. Mineralization results in a decline of the organic carbon pool *s*, with a fractional rate k_S_ (year^–1^). Moreover, a fraction of the unharvested biomass is degraded in the year of production (k_B_, year^–1^). The organic carbon in manure and unharvested biomass that remains undegraded after the year or production is added to *s*. A proportion of available inorganic N is converted to biomass and lost through volatilization, leaching, denitrification and runoff, resulting in a relative rate of withdrawal of inorganic nitrogen (k_W_, year^–1^). Nitrogen soil losses are calculated as the difference between plant uptake and total withdrawal (Fig. 2). The withdrawal fraction is assumed to be 95% of the available inorganic nitrogen *n* [9], which is applicable on well-drained soils in temperate regions where winter precipitation exceeds evapotranspiration resulting in complete loss of nitrate N [25]. Only small residues of inorganic N in NH_4_^+^ persist in winter when not subject to nitrification [26]. Parameters of soil processes are presented in Table S4.

**Figure 3:**
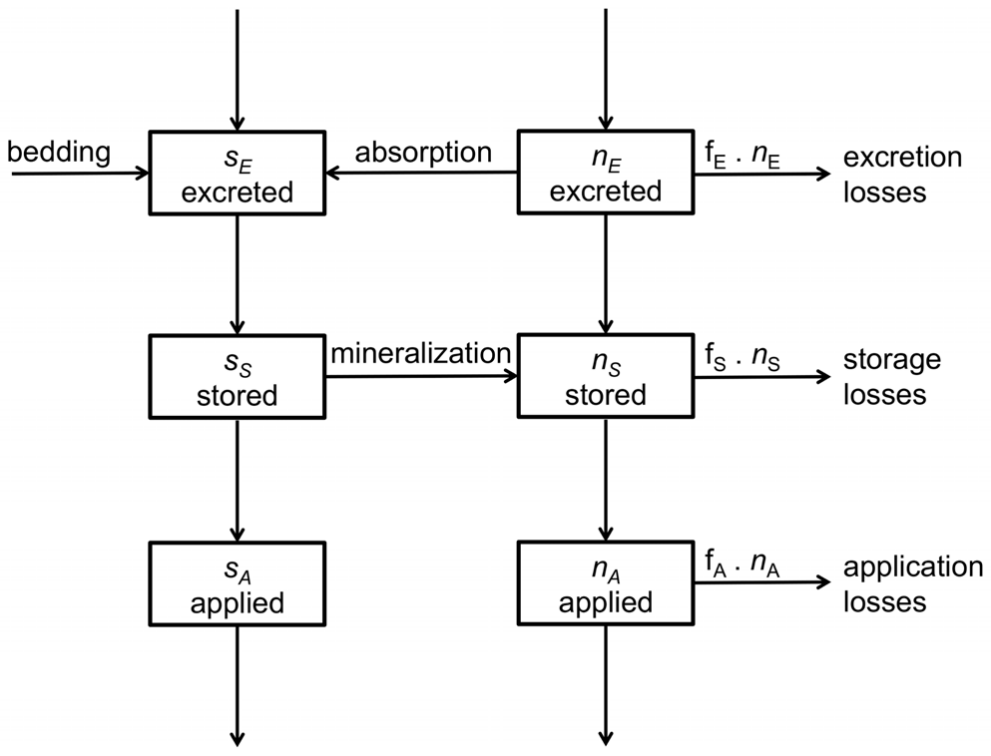
Conversions and losses of organic and inorganic nitrogen (*s* and *n*) in excreted cattle manure as affected by events and processes in the consecutive stages of the manure handling chain on a farm. The arrows indicate flows of nitrogen between the pools.

Nitrogen in animal excreta is present in organic and inorganic forms. An overview of the processes and conversions of N in manure is presented in Fig. 3. The calculations are largely based on calculation procedures presented by Dämmgen and Hutchings [27]; we followed the same steps in the calculations but adjusted the calculation of mineralization of manure organic N (see below). Part of the inorganic N can be adsorbed to straw and manure additives. The inorganic N is prone to emission by NH_3_ volatilization after excretion in the barn (f_E_, g g^–1^), during storage (f_S_, g g^–1^) and after application to the field (f_A_, g g^–1^). The loss fractions f_E_, f_S_ and f_A_ are dependent on the barn and storage conditions, and the method of manure application and extra emission mitigating measures after application such as irrigation or application during rainfall. The values of manure parameters as used in the model are presented in Table S5.

To estimate the mineralization or immobilization of N due to degradation of OM by microorganisms we use the following equation:
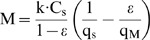
(5)Where

*M* = net mineralization (kg year^–1^).

*k* = fractional degradation rate of the substrate (year^–1^).

*C_S_* = amount of carbon in the substrate (kg C).

*ε* = growth efficiency of the microorganisms (kg kg^–1^).

*q_S_* = C:N ratio of the substrate (kg C kg^–1^ N).

*q_M_* = C:N ratio of the microorganisms (kg C kg^–1^ N).

Microorganisms break down the OM with apparent fractional degradation rate k. However, because microorganisms grow due to this degradation process with growth efficiency ε, this results in an addition to the OM, so the observed degradation rate should be corrected for their growth efficiency, and the true degradation rate is C_S_•k/(1–ε). The degradation of the OM is associated with release of N, determined by the C:N ratio of the substrate (q_S_). The micro-organisms will incorporate part or all of this N, dependent on their C:N ratio (q_M_). When the C:N ratio of the substrate is high, the release of N from OM is lower than the incorporation into microbial biomass, and as a consequence mineral N from the soil solution may be incorporated. This results in negative values for N release in the equation above, indicating net immobilization. Mineralization occurs when the C:N ratio of the substrate is lower than q_S_/ε.

The gross margin (revenues minus variable costs) was used as indicator for the economic performance of the farm. Gross margin is considered the most appropriate economic indicator in a fixed milk quota system [28] and is more sensitive to changes in farm management than total farm results, which also include fixed costs [29]. The revenues included returns from milk, meat and crop sales and other sources, and the variable costs were made for purchases of feeds and bedding material, veterinary care, breeding association and miscellaneous costs (Table S6).

### Case Study Farm and Scenarios

We defined a typical grassland-based organic dairy farm on a sandy soil in the province of Gelderland, The Netherlands. The farm area is 66 ha, of which 60 ha is grassland and 6 ha is used for cultivation of silage maize. The herd consists of 85 Holstein Frisian cows with a replacement rate of 25%. The herd is housed in a sloping floor barn, wherein a mixture of faeces, urine and bedding material (mostly wheat straw; SCM) is produced. The SCM is removed regularly from the barn and is stockpiled for storage. The animals graze during a period of 200 days per year (from mid April until the end of October) in a day-and-night grazing system wherein the cows spend 20 hours per day outdoors. The mown grass is conserved as silage and fed to cows indoors during the 165 days winter season.

We developed scenarios to evaluate the effects of changes in parameters due to adjustment in individual farm management practices regarding grassland and manure management, and a more integrated strategy combining various effective measures. With these scenarios we evaluated long-term productive, environmental and economic farm performance as affected by:

- Different animal housing systems that produce either SCM (faeces and urine mixed with wheat straw) from the sloping floor barn that is standard for the farm (scenario M), or slurry (mixed faeces and urine) from a cubicle housing system (scenario S). The latter results in lower fractional loss rates during storage and after application (f_S_ and f_A_) and higher yield of silage maize (Y_MAIZE_).
- Delayed mowing of grass (scenarios DS and DM) resulting in harvesting of more mature grass with lower feed quality (k_D,GRASS_) and N content (lower α_MIN,H_, α_MAX,H_ and α_MAX,T_) [9], [30], [31]. Due to the proportionality between feed quality and manure degradability, also k_M_ will decline in these scenarios.
- The use of the additives zeolite, lava meal and farm topsoil that are applied on SCM bedding inside the barn (scenarios MZ, ML and MT). The impacts of these additives on N losses after excretion in the barn, during storage and after application to the field and on N uptake and dry matter yield of grass and silage maize (at physiological maturity) have been quantified by [13], [14]. Emission factors f_E_, f_S_ and f_A_, grassland production (Fig. 2) and maize yields (Y_MAIZE_) were derived from these experimental results. Costs for additives were included in the price of bedding material.
- Alternative SCM storage systems of composting, roofing or covering by an impermeable sheet (scenarios MC, MR and MU). Shah et al. [32] quantified the consequences of these measures for emissions during storage (f_S_).
- Combining covering of the manure by an impermeable sheet (anaerobic storage) with 10 mm irrigation immediately after manure application to the field (scenario MUI). These combined measures affect NH_3_ volatilization after application (f_A_) and N recovery by the grassland (Fig. 2) as analysed experimentally by [15].
- A combination of measures of SCM handling that appeared most promising for productive, environmental and economic indicators from the previously described scenarios. This integrated strategy contained practices of delayed mowing, zeolite additive, covering with an impermeable sheet and irrigation after application (scenario DMZUI).

A complete overview of the parameters settings for all scenarios is provided in Table S7.

For the starting conditions for all scenarios we assumed the current situation on the farm, which has been under the management described above that is comparable to scenario M for almost 20 years, hence there is still build-up of soil organic matter and a steady state has not been reached. The long-term dynamics of the state variables *s*, *c* and *n* were evaluated for each of the scenarios for simulation duration of 200 years assuming constant farm management. Moreover, the productivity of the farm under the different scenarios was determined with the feed self-supply rate, which at the target level of animal outputs reflects the production of on-farm feeds (grass and silage maize). The farm gate N balance (inputs minus outputs in products) reflects the total farm N losses and was used as an indicator for environmental performance. The gross margin was the indicator of economic performance for each scenario.

## Results

### Slurry and SCM-based Systems

**Figure 4:**
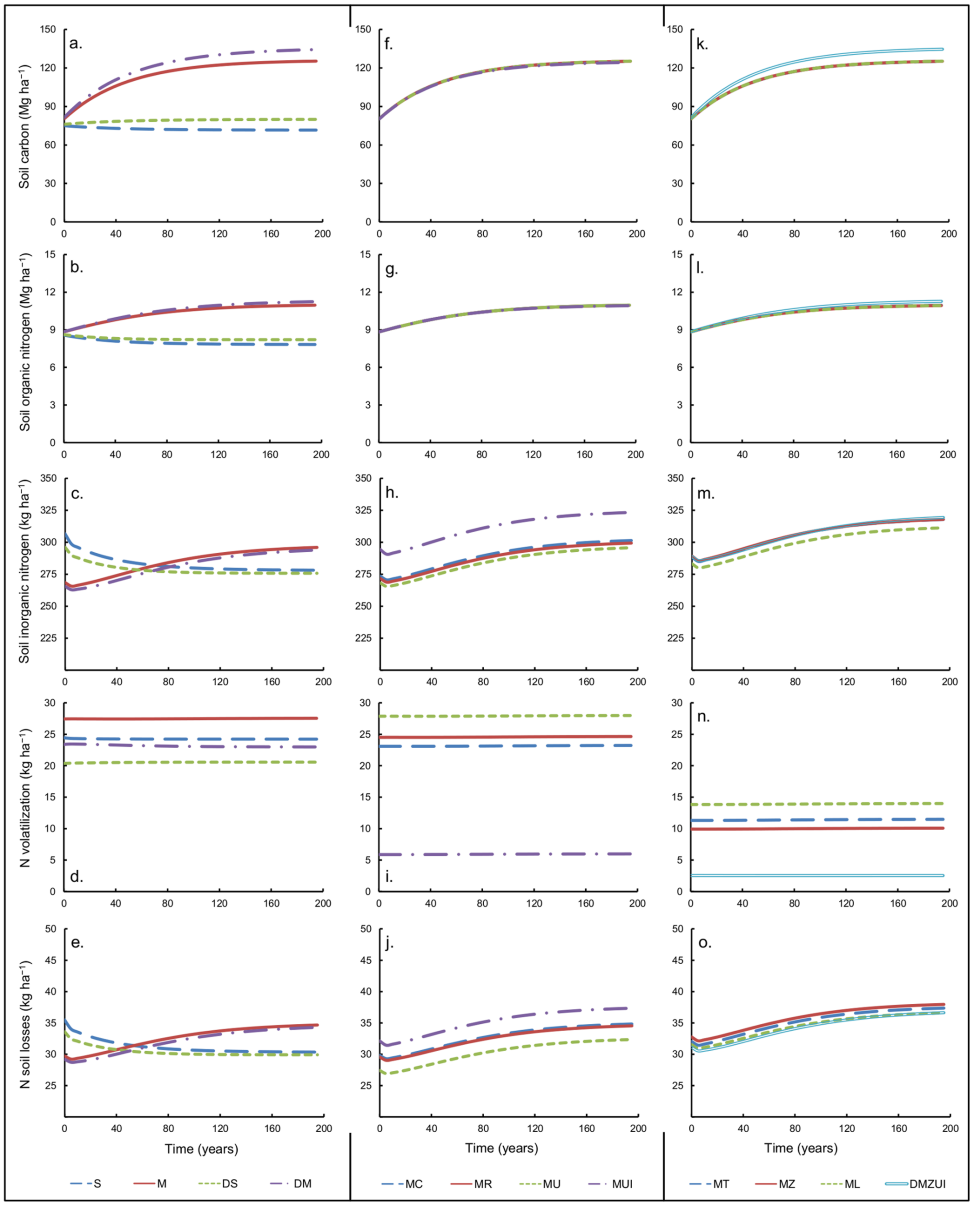
Dynamics of soil organic carbon (a, f, k) and nitrogen (b, g, l), inorganic nitrogen (c, h, m), N volatilization (d, i, n) and N soil losses (e, j, o) as affected by individual or combined management practices. Management scenarios were varied across columns: manure types (a-e), storage methods (f-j), and manure additives (k-o). Legends apply per column, with manure types (S = slurry, M = solid cattle manure), manure additives (T = farm topsoil, Z = zeolite, L = lava meal), storage methods (C = composting, R = roofed storage, U = impermeable sheet), I = irrigation, D = delayed mowing.

The slurry and SCM-based systems without mitigation measures in scenarios M and S contrasted strongly in C and N dynamics (Figs. 4a–4e). Initially the slurry-based system resulted in a larger amount of available inorganic N than the SCM-based system (Fig. 4c), which could support larger grassland productivity. However, the slurry-based system resulted in slightly declining soil organic C and N pools, whereas for the SCM system these pools gradually increased (Figs. 4a and 4b) due to the straw inputs for bedding. As a consequence, after 75 years of simulated management, the inorganic N availability was larger for the SCM system than for the slurry system (Fig. 4c), due to increased mineralization of the large organic N pool. This resulted in increased grassland production and a higher feed self-supply rate for the SCM-based system (Table 1). The NH_3_ emissions were higher from the SCM system (Fig. 4d). N soil losses were strongly linked to available inorganic N, therefore these soil losses from SCM system were initially lower than from the slurry-based system, but were larger than for the slurry system after 75 years (Fig. 4e). Thus, in the equilibrium situation total N losses (NH_3_ volatilization and N soil losses) were higher from the SCM-based system than from the slurry-based system (62 vs. 55 kg N ha^–1^ year^–1^).

**Table 1:** Effects of different manure types (slurry and solid cattle manure, SCM) and manure management practices on indicators of productive, environmental and economic farm performances.

Scenario	Feed self-supply	N-efficiency	Gross margin
	(%)	(%)	(€ ha^–1^)
Slurry, no treatments (S)	69	56	3130
SCM, no treatments (M)	74	52	2890
Slurry, delayed mowing (DS)	73	58	3174
SCM, delayed mowing (DM)	78	54	2940
SCM, composted (MC)	76	54	2936
SCM, roofed storage (MR)	75	53	2924
SCM, impermeable cover (sealed) (MU)	75	53	2910
SCM, sealed and irrigation (MUI)	82	61	3100
SCM, farm topsoil (MT)	80	58	3054
SCM, zeolite (MZ)	80	58	2960
SCM, lava meal (ML)	79	57	2872
SCM, combined treatments* (DMZUI)	85	63	3140

* Delayed mowing, use of zeolite manure additive, storage under an impermeable cover (sealed), and irrigation after application.

### Effects of Delayed Mowing

Later mowing of grass results in the on-farm production of more grassland biomass with a lower N content in scenarios DM and DS. This led to reductions of N volatilization of ca. 4.5 kg N ha^–1^ year^–1^ (Fig. 4d), while N soil losses also declined slightly (Fig. 4e). In the long term the simulated accumulation of soil organic C and N was larger when more mature grass of lower degradability was fed, both in slurry and SCM-based systems (Figs. 4a and 4b).

### Effects of Storage Measures and Irrigation

The storage treatments for SCM-based systems of roofing (scenario MR) or sealing with an impermeable sheet (MU) of the manure helped to reduce NH_3_ volatilization losses, although the reductions of losses during storage were largely compensated by extra emissions after application, in particular for the sealing treatment (Fig. 4i). The treatments had no effects on soil organic C and N dynamics (Figs. 4f and 4g), feed self-supply, whole farm N-efficiency and gross margin (Table 1). However, when sealing was combined with irrigation in scenario MUI, the application losses were avoided, and N volatilization could be reduced to only 6 kg N ha^–1^ year^–1^. As a compensation loss for lower volatilization, the combined treatment of sealing and irrigation caused higher N soil losses (Fig. 4j). Nevertheless, the inorganic N availability was larger due to sealing and irrigation (Fig. 4 h), resulting in better grassland production so that the productive, environmental and economic indicators of feed self-supply rate, farm N-efficiency and gross margin of scenario MUI were better than untreated SCM or only storage measures (Table 1).

### Effects of Bedding Additives

The addition of farm topsoil, zeolite or lava meal to the manure bedding (scenarios MT, MZ and ML) resulted in reduced emission through volatilization, which was proportionally but only partly compensated by more N soil losses, so that inorganic N availability was higher (Figs. 4m–4o). These effects were strongest for the zeolite additive. The three additives resulted in similar improvements of feed self-supply rate, farm N-efficiency and gross margin when compared to the SCM-based system without treatments (Table 1). There were no differences among the additives on the long-term soil C and N dynamics (Figs. 4k, 4l).

### Combined Effects in a Coherent Strategy

**Figure 5:**
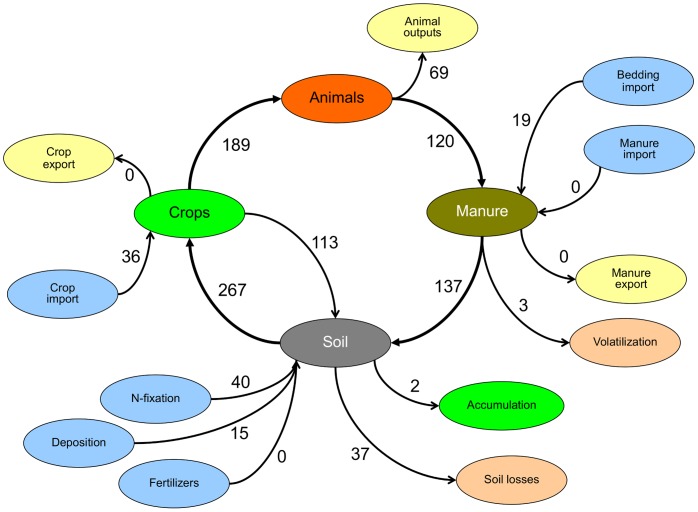
Nitrogen flows (kg N/ha/year) for a dairy farm in steady state with delayed mowing of grassland, producing SCM and using zeolite as bedding additive, manure storage under an impermeable sheet, and irrigation after application (scenario DMZUI in Fig. **4).**

A strategy of emission mitigation was defined by combining the most successful practices for management of SCM: delayed mowing of grassland, using zeolite as bedding additive, storage under an impermeable sheet, and irrigation after application (scenario DMZUI). This strategy reduced NH_3_ volatilization with limited compensatory N soil losses, so that total losses were reduced to 40 kg N ha^–1^ year^–1^ and inorganic N availability was enhanced (Figs. 4k–4o; Fig. 5). Also the long-term increments in soil C and N were larger than for untreated SCM due to the lower degradability of mature grass after delayed mowing. Both the feed self-supply rate and the whole farm N efficiency were superior to all alternative systems, and the gross margin was comparable to that of the slurry-based systems (Table 1).

## Discussion

SCM-based systems are often associated with larger N losses than slurry-based systems [33]. Many of these losses seem to be avoidable in the short term through appropriate management practices for manure after excretion in the barn and during storage as observed in experiments. These practices could be evaluated in the model simulations for their impacts on N losses and soil C and N pools in the long term:

- Delayed mowing results in a higher C:N ratio in the feed, and more organic C and less NH3 in the manure [10], [21]. The high C:N ratio of the manure OM may cause immobilization of N upon application to soil, followed by a slow rate of mineralization [34]. Simulation results revealed that this practice contributes to increased soil OM build-up, but has no long-term effects on N losses.
- The application of additives like zeolite, farm soil or lava meal to the bedding material results in increased NH_4_^+^ adsorption, which reduces NH_3_ volatilization [35]–[37]. The model outputs showed that this will result in some compensatory soil losses, but soil N availability will improve in a long run. Soil organic C and N pools were not affected when compared to SCM system without any treatment.
- Covering manure heaps with an impermeable sheet creates a physical barrier that avoids exposure to air and prevents NH_3_ diffusion to the atmosphere [38]–[40]. This only affects the storage phase, but after application to the field the simulated volatilization losses were higher.
- Moreover, covering results in anaerobic conditions that slow down OM degradation during storage [34], [41], but the labile OM will be rapidly degraded after application to the field. Thus no effects on soil organic C and N pools can be expected, as reflected in the simulation results.

Thus, the simulations demonstrated that individual mitigation measures to reduce losses resulted in compensatory loss pathways. Moreover, in contrast to slurry, SCM cannot be injected in grasslands, while shallow injection of cattle slurry can reduce NH_3_ volatilization by up to 74% [42], [43]. Conserving inorganic N in the manure during the housing and storage phases leads to higher concentrations in applied manure, and can result in increased emission rates during surface application. Consequently, for SCM additional measures like irrigation or application shortly before rainfall are needed after application to the field to enhance infiltration of total ammoniacal N into the soil [15]. Therefore, for effective mitigation of N losses at the farming systems level and in the long term, a strategy composed of a series of techniques would be needed to address the various potential outflows of N from the system. The model simulations for scenario DMZUI demonstrated that combined management practices of applying zeolite on the SCM bedding inside the barn, anaerobic storage of this manure under impermeable plastic sheet, 10 mm of irrigation immediately after surface application of the manure on grassland and its delayed mowing is the most effective combination to increase soil C and N stocks and to reduce N losses. Such a strategy could result in lower losses, higher productivity and similar economic results as slurry-based systems.

A large advantage of SCM-based systems in the long term is the increased soil organic C and organic N contents (Figs. 4a and 4b) as compared to slurry-based systems due to larger inputs of OM. For SCM scenarios, annual rate of increase in soil OM was greatest in the early phases of the simulation and very low near the end as the soil approached an equilibrium state. This is in agreement with findings in a long-term simulation study [44] and experimental data [45], [46]. Besides the contribution to C sequestration, increasing the OM content of soils is important for physical and biological soil properties and processes that support many ecosystem functions. OM contributes to the water holding capacity, cation exchange capacity and infiltration capacity of soils [47], [48]. Moreover, there is in general a positive relationship between soil C content and soil microbial biomass [49], and any practice that increases the amount of soil OM improves its biological activity, e.g. [50]. These biota activities can enhance mineralization of soil OM, and hence the supply of inorganic N for plant growth.

In modelling we search for a balance between the level of detail, the precision required, the model’s flexibility and the data requirements [51]–[53]. The Farm DANCES model used in this study can be characterized as an eco-mathematical summary model that quantifies the dynamics of organic N and C, and inorganic N, as an instrument to evaluate management strategies. By combining all the relevant processes in the farm N cycle, the model allows to assess interactions among these processes and to identify emergent system properties such as compensatory loss pathways. It offers a quantitative framework for evaluating both short-term and long-term effects of management interventions aimed at improving nutrient use efficiency [54]. This framework supports formulation of scenarios describing future developments, rather than exact prediction (cf. [55]). We are not aware of any empirical data that would enable validation of the whole farm model over a substantial period of time of 25 years or more. Therefore, model validity must be inferred from validity of its components and the plausibility of its results. The model constitutes a complement to studies that emphasize short-term optimization of performance of farm system components, such as emission from barns or N leaching at given soil management, and studies that focus on empirical relations between production factors, such as fertilizer and outputs [9].

From a model user’s perspective, the focus of the Farm DANCES model is on quantifying interactions among farm components and biological processes and to provide insight into these interactions to its users, which are currently predominantly researchers and students. The model builds on existing knowledge of biological processes, is data-sparse, can be parameterized with experimental data, and the graphical user interface of the model is intuitive and easy to use. There is scope to improve the model by adding flexibility to the scenarios over time, by including the impact of varying environmental conditions (e.g. temperature and moisture dependence of degradation processes), by further specification of N soil loss pathways and greenhouse gas emissions (e.g. [56]), and by compartmentalizing soil organic matter pools with distinctive degradation dynamics (e.g. [57]). However, these extensions would sacrifice the insightfulness, whereas various modelling studies have demonstrated that relatively simple dynamic models that are based on the correct process representation and data can be extremely accurate and useful (e.g., for soil processes: [58]–[60]; for plant growth: [61], [62]; review for livestock systems: [20]).

### Conclusions

The simulation results demonstrated that individual emission mitigation measures were often insufficient to reduce N losses at the farming systems level. Practices that reduced NH_3_ emissions from animal excreta in the barn or during storage resulted in larger losses after application of manure to the field, through either volatilization of NH_3_ or soil losses, i.e. the aggregated flows of runoff, leaching and denitrification. The integrated strategy combining the most effective practices resulted in build-up of soil organic C and N pools, sufficient nutrient availability for plants and low emission rates. This strategy of combined grassland and manure management practices included delayed mowing of grass and fertilization with solid cattle manure that is treated with zeolite, stored under an impermeable sheet and irrigated after application. This strategy can reduce losses to the environment, improve soil properties by larger organic C and N stocks, and increase availability of N for plants uptake, grassland productivity, enhance the feed self-supply. We conclude that SCM-based systems employing a coherent strategy of manure utilization practices can contribute to improved productive, environmental and economic performance of dairy farming systems.

## Supporting Information

Table S1 **Animal parameters.**
(DOCX)

Table S2 **Crop parameters.**
(DOCX)

Table S3 **Feed parameters.**
(DOCX)

Table S4 **Soil parameters.**
(DOCX)

Table S5 **Manure parameters.**
(DOCX)

Table S6 **Economic parameters.**
(DOCX)

Table S7 **Scenario parameters: adjusted parameters per scenario compared to the standard SCM (M).**
(DOCX)

## References

[1] Sutton MA, Oenema O, Erisman JW, Leip A, Van Grinsven H, et al (2011) Too much of a good thing. Nature 472: 159–161. 21478874 10.1038/472159a

[2] Matson PA, Naylor R, Ortiz-Monasterio I (1998) Integration of environmental, agronomic, and economic aspects of fertilizer management. Science 280: 112–115. 9525856 10.1126/science.280.5360.112

[3] Galloway JN, Aber JD, Erisman JW, Seitzinger SP, Howarth RW, et al (2003) The nitrogen cascade. BioScience 53: 341–356.

[4] Watson CA, Öborn I, Eriksen J, Edwards AC (2005) Perspectives on nutrient management in mixed farming systems. Soil Use Manage 21: 132–140.

[5] Herrero M, Thornton PK, Notenbaert AM, Wood S, Msangi S, et al (2010) Smart investments in sustainable food production: revisiting mixed crop-livestock systems. Science 327: 822–825. 20150490 10.1126/science.1183725

[6] Küstermann B, Christen O, Hülsbergen K-J (2010) Modelling nitrogen cycles of farming systems as basis of site- and farm-specific nitrogen management. Agric Ecosyst Environ 135: 70–80.

[7] Groot JCJ, Oomen GJM, Rossing WAH (2012) Multi-objective optimization and design of farming systems. Agric Syst 110: 63–77.

[8] Rufino MC, Hengsdijk H, Verhagen A (2009) Analysing integration and diversity in agro-ecosystems by using indicators of network analysis. Nutr Cycl Agroecosyst 84: 229–247.

[9] Groot JCJ, Rossing WAH, Lantinga EA, Van Keulen H (2003) Exploring the potential for improved internal nutrient cycling in dairy farming systems, using an eco-mathematical model. NJAS – Wageningen J Life Sci 51: 165–194.

[10] Groot JCJ, Rossing WAH, Lantinga EA (2006) Evolution of farm management, nitrogen efficiency and economic performance on Dutch dairy farms reducing external inputs. Livest Sci 100: 99–110.

[11] Watson CA, Atkinson D, Gosling P, Jackson LR, Rayns FW (2002) Managing soil fertility in organic farming systems. Soil Use Manage 18: 239–247.

[12] Henkens PLCM, Van Keulen H (2001) Mineral policy in the Netherlands and nitrate policy within the European Community. Neth J Agric Sci 49: 117–134.

[13] Shah GA, Shah GM, Groot JCJ, Groot Koerkamp PWG, Lantinga EA (2013) Effects of bedding additives on carbon and nitrogen losses from a sloping-floor cattle-housing system. J Agric Sci, in press.

[14] Shah GA, Shah GM, Groot JCJ, Lantinga EA (2013) Bedding additives reduce nitrogen losses and improve fertilizer value of cattle straw manure. Nutr Cycl Agroecosyst, in press.

[15] Shah GM, Shah GA, Groot JCJ, Oenema O, Lantinga EA (2012) Irrigation and lava meal use reduce ammonia emission and improve N utilization when solid cattle manure is applied to grassland Agric Ecosyst Environ. 160: 59–65.

[16] Rotz CA (2004) Management to reduce nitrogen losses in animal production. J Anim Sci 82: E119–E137. 15471791 10.2527/2004.8213_supplE119x

[17] Amon B, Kryvoruchko V, Amon T, Zechmeister-Boltenstern S (2006) Methane, nitrous oxide and ammonia emissions during storage and after application of dairy cattle slurry and influence of slurry treatment. Agric Ecosyst Environ 112: 153–162.

[18] Rotz CA, Oenema J, Van Keulen H (2006) Whole farm management to reduce nutrient losses from dairy farms: a simulation study. Appl Eng Agric 22: 773–784.

[19] Rotz CA, Taube F, Russelle M P, Oenema J, Sanderson MA, et al (2005) Whole-farm perspectives of nutrient flows in grassland agriculture. Crop Sci 45: 2139–2159.

[20] Gouttenoire L, Cournut S, Ingrand S (2011) Modelling as a tool to redesign livestock farming systems: a literature review Animal 1–15. 10.1017/S175173111100111X 22440473

[21] Reijs JW, Sonneveld MPW, Sørensen P, Schils RLM, Groot JCJ, et al (2007) Utilization of nitrogen from cattle slurry applied to grassland as affected by diet composition. Agric Ecosyst Environ 118: 65–78.

[22] Van Es AJH (1975) Feed evaluation for dairy cows. Livest Prod Sci 2: 95–107.

[23] Tamminga S, Van Straalen WM, Subnel APJ, Meijer RGM, Steg A, et al (1994) The Dutch protein evaluation system: the DVE/OEB-system Livest Prod Sci. 40: 139–155.

[24] Goudriaan J, Monteith JL (1990) A mathematical function for crop growth based on light interception and leaf area expansion. Ann Bot 66: 695–701.

[25] Scholefield D, Lockyer DR, Whitehead DC, Tyson KC (1991) A model to predict transformations and losses of nitrogen in UK pastures grazed by beef cattle. Plant Soil 132: 165–177.

[26] Whitehead DC (1995) Grassland Nitrogen CAB International, Wallingford, 397 pp.

[27] Dämmgen U, Hutchings N J (2008) Emissions of gaseous nitrogen species from manure management: a new approach. Environ Pollut 154: 488–497. 17562350 10.1016/j.envpol.2007.03.017

[28] Rougoor CW, Dijkhuizen AA, Huirne RBM, Mandersloot F, Schukken YH (1997) Relationships between technical, economic and environmental results on dairy farms: an explanatory study. Livest Prod Sci 47: 235–244.

[29] Ondersteijn CJM, Beldman ACG, Daatselaar CHG, Giessen GWJ, Huirne RBM (2003) Farm structure and farm management: effective ways to reduce nutrient surpluses on dairy farms and their financial impacts. Livest Prod Sci 84: 171–181.

[30] Groot JCJ, Neuteboom JH (1997) Composition and digestibility during ageing of Italian ryegrass leaves of consecutive insertion levels. J Sci Food Agric 75: 227–236.

[31] Groot JCJ, Lantinga EA (2004) An object-oriented model of the morphological development and digestibility of perennial ryegrass. Ecol Model 177: 297–312.

[32] Shah GM, Shah GA, Groot JCJ, Oenema O, Lantinga EA (2013) Magnitude and routes of nitrogen and carbon losses from solid cattle manure subjected to various storage conditions. Nutr Cycl Agroecosyst, in press.

[33] Mosquera J, Hol JMG, Monteny GJ (2006) Gaseous emissions from a deep litter farming system for dairy cattle. In: International Congress Series (Eds CR Soliva, J Takahashi, M Kreuzer), Vol 1293, 291–294.

[34] Kirchmann H, Witter E (1989) Ammonia volatilization during aerobic and anaerobic manure decomposition. Plant Soil 115: 35–41.

[35] Mumpton FA, Fishman PH (1977) The application of natural zeolites in animal science and aquaculture. J Anim Sci 45: 1188–1203.

[36] Witter E, Lopez-Real J (1988) Nitrogen losses during the composting of sewage sludge, and the effectiveness of clay soil, zeolite, and compost in adsorbing the volatilized ammonia. Biol Waste 23: 279–294.

[37] Ndegwa PM, Hristov AN, Arogo J, Sheffield RE (2008) A review of ammonia emission mitigation techniques for concentrated animal feeding operations. Biosyst Eng 100: 453–469.

[38] Kirchmann H (1985) Losses, plant uptake and utilization of manure nitrogen during a production cycle. Acta Agric Scand Suppl 24: 1–77.

[39] Hansen MN, Henriksen K, Sommer SG (2006) Observations of production and emission of greenhouse gases and ammonia during storage of solids separated from pig slurry: Effects of covering. Atmos Environ 40: 4172–4181.

[40] Shah GM, Groot JCJ, Oenema O, Lantinga EA (2012) Covered storage reduces losses and improves crop utilisation of nitrogen from solid cattle manure. Nutr Cycl Agroecosyst 94: 299–312.

[41] Thomsen IK, Olesen JE (2000) C and N mineralization of composted and anaerobically stored ruminant manure in differently textured soils. J Agric Sci 135: 151–159.

[42] Misselbrook TH, Smith KA, Johnson RA, Pain BF (2002) Slurry application techniques to reduce ammonia emissions: Results of some UK field-scale experiments. Biosyst Eng 81: 313–321.

[43] Hansen MN, Sommer SG, Madsen NP (2003) Reduction of ammonia emission by shallow slurry injection: Injection efficiency and additional energy demand. J Environ Qual 32: 1099–1104. 12809311 10.2134/jeq2003.1099

[44] Foereid B, Høgh-Jensen H (2004) Carbon sequestration potential of organic agriculture in northern Europe - A modelling approach. Nutr Cycl Agroecosyst 68: 13–24.

[45] Jackman RH (1964) Accumulation of organic matter in some New Zealand soils under permanent pasture. NZ J Agric Res 7: 445–471.

[46] Johnston AE, Poulton PR, Coleman K (2009) Soil organic matter: its importance in sustainable agriculture and carbon dioxide fluxes. Adv Agron 101: 1–57.

[47] Haynes RJ, Naidu R (1998) Influence of lime, fertilizer and manure applications on soil organic matter content and soil physical conditions: a review. Nutr Cycl Agroecosyst 51: 123–137.

[48] Bronick CJ, Lal R (2005) Soil structure and management: a review. Geoderma 124: 3–22.

[49] Dick RP (1992) A review: long term effects of agricultural systems on soil biochemical and microbial parameters. Agric Ecosyst Environ 40: 25–36.

[50] Rashid MI, de Goede RGM, Brussaard L, Bloem J, Lantinga EA (2013) Production-ecological modelling explains differences between potential soil N mineralisation and actual herbage N uptake. Ecol Appl, in press.

[51] Brooks RJ, Tobias AM (1996) Choosing the best model: level of detail, complexity, and model performance. Math Comput Model 24: 1–14.

[52] Thornton PK, Herrero M (2001) Integrated crop–livestock simulation models for scenario analysis and impact assessment. Agric Syst 70: 581–602.

[53] Astrup R, Coates KD, Hall E (2008) Finding the appropriate level of complexity for a simulation model: An example with a forest growth model. For Ecol Manage 256: 1659–1665.

[54] Van Apeldoorn DF, Kok K, Sonneveld MPW, Veldkamp TA (2011) Panarchy rules: Rethinking resilience of agroecosystems, evidence from Dutch dairy farming. Ecol Soc 16 (1).

[55] Carpenter SR (2002) Ecological futures: Building an ecology of the long now. Ecology 83: 2069–2083.

[56] Schils RLM, Verhagen A, Aarts HFM, Šebek LBJ (2005) A farm level approach to define successful mitigation strategies for GHG emissions from ruminant livestock systems. Nutr Cycl Agroecosyst 71: 163–175.

[57] Tipping E, Rowe EC, Evans CD, Mills RTE, Emmett BA, et al (2012) N14C: A plant–soil nitrogen and carbon cycling model to simulate terrestrial ecosystem responses to atmospheric nitrogen deposition. Ecol Model 247: 11–26.

[58] Grace P, Ladd J, Robertson G, Gage S (2006) SOCRATES–A simple model for predicting long-term changes in soil organic carbon in terrestrial ecosystems. Soil Biol Biochem 38: 1172–1176.

[59] Kemanian A R, Stöckle C O (2010) C-Farm: A simple model to evaluate the carbon balance of soil profiles. Eur J Agron 32: 22–29.

[60] Saffih-Hdadi K, Mary B (2008) Modeling consequences of straw residues export on soil organic carbon. Soil Biol Biochem 40: 594–607.

[61] Van der Werf W, Keesman K, Burgess P, Graves A, Pilbeam D, et al (2007) Yield-SAFE: A parameter-sparse, process-based dynamic model for predicting resource capture, growth, and production in agroforestry systems. Ecol Engineer 29: 419–433.

[62] Romera AJ, Beukes P, Clark C, Clark D, Levy H, et al (2010) Use of a pasture growth model to estimate herbage mass at a paddock scale and assist management on dairy farms. Comput Electron Agric 74: 66–72.

