# Supplementary material for: Simulation of Long-Term Carbon and Nitrogen Dynamics in Grassland-Based Dairy Farming Systems to Evaluate Mitigation Strategies for Nutrient Losses
Source: PLoS One. 2013 Jun 27;8(6):e67279. doi: 10.1371/journal.pone.0067279 (PMC3694978; doi:10.1371/journal.pone.0067279)
Supplement: Table S1 — Animal parameters. (DOCX) [file pone.0067279.s001.docx]

Table S1. Animal parameters.

| **Parameter** | **Value** | **Unit** |
| --- | --- | --- |
| 1.1 Number of milk cows | 85 | - |
| 1.2 Replacement rate | 25 | % year^–1^ |
| 1.3 Weight of adult cow | 650 | kg |
| 1.4 Carcass percentage | 55 | % |
| 1.5 Milk production | 22 | kg day^–1^ |
| 1.6 Milk protein content | 3.8 | % |
| 1.7 Milk fat content | 4.8 | % |
| 1.8 Proportion of the year spent grazing | 45 | % |
