# Supplementary material for: Simulation of Long-Term Carbon and Nitrogen Dynamics in Grassland-Based Dairy Farming Systems to Evaluate Mitigation Strategies for Nutrient Losses
Source: PLoS One. 2013 Jun 27;8(6):e67279. doi: 10.1371/journal.pone.0067279 (PMC3694978; doi:10.1371/journal.pone.0067279)
Supplement: Table S2 — Crop parameters. (DOCX) [file pone.0067279.s002.docx]

Table S2. Crop parameters.

| **Parameter** | **Value** | **Unit** |
| --- | --- | --- |
| *Silage maize (6 ha):* |  |  |
| 2.1 Dry matter (DM) yield (Y_MAIZE_) | 14241 | kg DM ha^–1^ |
| 2.2 Residue yield ratio | 0.15 | kg kg^–1^ |
|  |  |  |
| *Grassland (60 ha):* |  |  |
| 2.3 Symbiotic N fixation | 40 | kg N ha^–1^ |
| 2.4 Intercept for nitrogen uptake | 70 | kg N ha^–1^ |
| 2.5 Maximum nitrogen uptake in harvestable biomass (U_MAX_) | 430 | kg N ha^–1^ |
| 2.6 Initial response of uptake in harvestable biomass to available N (ρ_H_) | 0.75 | kg kg^–1^ |
| 2.7 Minimum nitrogen content in harvestable biomass (α_MIN,H_) | 18 | g N kg^–1^ DM |
| 2.8 Maximum nitrogen content in harvestable biomass (α_MAX,H_) | 32 | g N kg^–1^ DM |
| 2.9 Decline in response to available N (λ) | 0.015 | kg kg^–1^ |
| 2.10 Harvested N as a proportion of total N uptake (*h*_N_) | 0.76 | kg kg^–1^ |
| 2.11 Initial response of uptake in total biomass to available N (ρ_T_) | 0.75 | kg kg^–1^ |
| 2.12 Minimum nitrogen content in total biomass (α_MIN,T_) | 10 | g N kg^–1^ DM |
| 2.13 Maximum nitrogen content in total biomass (α_MAX,T_) | 25 | g N kg^–1^ DM |
