# Supplementary material for: Simulation of Long-Term Carbon and Nitrogen Dynamics in Grassland-Based Dairy Farming Systems to Evaluate Mitigation Strategies for Nutrient Losses
Source: PLoS One. 2013 Jun 27;8(6):e67279. doi: 10.1371/journal.pone.0067279 (PMC3694978; doi:10.1371/journal.pone.0067279)
Supplement: Table S3 — Feed parameters. (DOCX) [file pone.0067279.s003.docx]

Table S3. Feed parameters.

| **Parameter** | **Value** | **Unit** |
| --- | --- | --- |
| *Silage maize:* |  |  |
| 3.1 Dry matter digestibility (k_D,MAIZE_) | 800 | g kg^–1^ |
| 3.2 Feeding losses | 50 | g kg^–1^ |
| 3.3 Nitrogen content | 12 | g N kg^–1^ DM |
|  |  |  |
| *Grass:* |  |  |
| 3.4 Dry matter digestibility (k_D,GRASS_) | 830 | g kg^–1^ |
| 3.5 Feeding losses | 100 | g kg^–1^ |
|  |  |  |
| *Supplement:* |  |  |
| 3.6 Dry matter digestibility (k_D,SUPPL_) | 750 | g kg^–1^ |
| 3.7 Feeding losses | 50 | g kg^–1^ |
| 3.8 Nitrogen content | 20 | g N kg^–1^ DM |
