# Supplementary material for: Simulation of Long-Term Carbon and Nitrogen Dynamics in Grassland-Based Dairy Farming Systems to Evaluate Mitigation Strategies for Nutrient Losses
Source: PLoS One. 2013 Jun 27;8(6):e67279. doi: 10.1371/journal.pone.0067279 (PMC3694978; doi:10.1371/journal.pone.0067279)
Supplement: Table S4 — Soil parameters. (DOCX) [file pone.0067279.s004.docx]

Table S4. Soil parameters.

| **Parameter** | **Value** | **Unit** |
| --- | --- | --- |
| 4.1 Depth | 30 | cm |
| 4.2 Bulk density | 1.45 | g cm^–3^ |
| 4.3 Initial organic matter content | 30 | g kg^–1^ |
| 4.4 Initial total nitrogen content | 2 | g kg^–1^ |
| 4.5 Fractional withdrawal rate of inorganic nitrogen (k_W_) | 0.95 | year^–1^ |
| 4.6 Fractional degradation rate of unharvested biomass (k_B_) | 0.80 | year^–1^ |
| 4.7 Fractional degradation rate of soil organic matter (k_S_) | 0.02 | year^–1^ |
| 4.8 Microbial efficiency (ε) | 0.30 | kg kg^–1^ |
| 4.9 C:N ratio of microorganisms (q_M_) | 11.3 | kg C kg^–1^ N |
