# Supplementary material for: Simulation of Long-Term Carbon and Nitrogen Dynamics in Grassland-Based Dairy Farming Systems to Evaluate Mitigation Strategies for Nutrient Losses
Source: PLoS One. 2013 Jun 27;8(6):e67279. doi: 10.1371/journal.pone.0067279 (PMC3694978; doi:10.1371/journal.pone.0067279)
Supplement: Table S5 — Manure parameters. (DOCX) [file pone.0067279.s005.docx]

Table S5. Manure parameters.

| **Parameter** | **Value** | **Unit** |
| --- | --- | --- |
| 5.1 Fractional emission rate of inorganic N after excretion (f_E_) | 0.16 | g g^–1^ |
| 5.2 Fractional emission rate of inorganic N during storage (f_S_) | 0.35 | g g^–1^ |
| 5.3 Fractional emission rate of inorganic N after application (f_A_) | 0.40 | g kg^–1^ |
| 5.4 Supply of wheat straw for bedding (S_BED_) | 5 | kg LU^–1^ |
| 5.5 Maximum absorption of inorganic N by bedding (A_BED_) | 15.0 | g N kg^–1^ DM |
| 5.6 Carbon content of wheat straw | 448 | g C kg^–1^ DM |
| 5.7 Nitrogen content of wheat straw | 5.5 | g N kg^–1^ DM |
| 5.8 Proportionality constant of manure degradability and feed digestibility (g_M_) | 0.5 | - |
