# Supplementary material for: Simulation of Long-Term Carbon and Nitrogen Dynamics in Grassland-Based Dairy Farming Systems to Evaluate Mitigation Strategies for Nutrient Losses
Source: PLoS One. 2013 Jun 27;8(6):e67279. doi: 10.1371/journal.pone.0067279 (PMC3694978; doi:10.1371/journal.pone.0067279)
Supplement: Table S6 — Economic parameters. (DOCX) [file pone.0067279.s006.docx]

Table S6. Economic parameters.

| **Parameter** | **Value** | **Unit** |
| --- | --- | --- |
| 6.1 Milk price | 0.39 | € kg^–1^ |
| 6.2 Meat price | 1.00 | € g^–1^ |
| 6.3 Grass price for exported herbage | 0.08 | € kg^–1^ DM |
| 6.4 Other returns for the farm | 0 | € |
| 6.5 Price of supplementary feeds | 0.22 | € kg^–1^ DM |
| 6.6 Price of bedding material | 0.12 | € kg^–1^ DM |
| 6.7 Costs for breeding | 60.4 | € cow^–1^ |
| 6.8 Costs for veterinary care | 0.45 | € 100 kg^–1^ milk cow^–1^ |
| 6.9 Other animal costs | 35.5 | € cow^–1^ |
| 6.10 Costs for silage maize cultivation | 1261 | € ha^–1^ |
