# Supplementary material for: Simulation of Long-Term Carbon and Nitrogen Dynamics in Grassland-Based Dairy Farming Systems to Evaluate Mitigation Strategies for Nutrient Losses
Source: PLoS One. 2013 Jun 27;8(6):e67279. doi: 10.1371/journal.pone.0067279 (PMC3694978; doi:10.1371/journal.pone.0067279)
Supplement: Table S7 — Scenario parameters: adjusted parameters per scenario compared to the standard SCM (M). (DOCX) [file pone.0067279.s007.docx]

Table S7. Scenario parameters: adjusted parameters per scenario compared to the standard SCM (M).

S = slurry

DS = slurry, delayed mowing

DM = SCM, delayed mowing

MC = SCM, composted

MR = SCM, roofed storage

MU = SCM, covered with impermeable sheet

MUI = SCM, covered with impermeable sheet and irrigated

MT = SCM, farm top soil added to bedding

MZ = SCM, zeolite added to bedding

ML = SCM, lava meal added to bedding

DMZUI = SCM, delayed mowing, zeolite added to bedding, covered with impermeable sheet and irrigated

| **Parameter** | **S** | **DS** | **DM** | **MC** | **MR** | **MU** | **MUI** | **MT** | **MZ** | **ML** | **DMZUI** |
| --- | --- | --- | --- | --- | --- | --- | --- | --- | --- | --- | --- |
| 2.1 Y_MAIZE_ | 15000 | 15000 |  | 14612 | 14681 | 15275 | 15275 | 14857 | 14536 | 15275 | 15275 |
| 2.7 α_MIN,H_ |  | 15 | 15 |  |  |  |  |  |  |  | 15 |
| 2.8 α_MAX,H_ |  | 28 | 28 |  |  |  |  |  |  |  | 28 |
| 2.13 α_MAX,T_ |  | 23 | 23 |  |  |  |  |  |  |  | 23 |
| 3.4 k_D,GRASS_ |  | 750 | 750 |  |  |  |  |  |  |  | 750 |
| 5.1 f_E_ |  |  |  |  |  |  |  | 0.08 | 0.04 | 0.06 | 0.04 |
| 5.2 f_S_ | 0.15 | 0.15 |  | 0.33 | 0.12 | 0.00 | 0.00 | 0.21 | 0.18 | 0.21 | 0.00 |
| 5.3 f_A_ | 0.13 | 0.13 |  | 0.30 |  | 0.50 | 0.04 | 0.10 | 0.10 | 0.17 | 0.04 |
| 5.4 S_BED_ | 0 | 0 |  |  |  |  |  |  |  |  |  |
| 5.5 A_BED_ |  |  |  |  |  |  |  | 15.1 | 16.3 | 15.3 | 16.3 |
